# Recuperating lung decoction attenuates inflammation and oxidation in cigarette smoke‐induced COPD in rats via activation of ERK and Nrf2 pathways

**DOI:** 10.1002/cbf.3273

**Published:** 2017-07-27

**Authors:** Chunlei Li, Yue Yan, Qi Shi, Yanhua Kong, Longxia Gao, Haipeng Bao, Youlin Li

**Affiliations:** ^1^ Beijing University of Chinese Medicine Beijing China; ^2^ The Key Institute of State Administration of Traditional Chinese Medicine (pneumonopathy chronic cough and dyspnea) Beijing Key Laboratory (No.BZ0321) China‐Japan Friendship Hospital Beijing China

**Keywords:** COPD, ERK/Nrf2 signalling pathway, inflammation, oxidative stress

## Abstract

Oxidative/antioxidative imbalance and chronic inflammation are the main contributors to the pathogenesis of chronic obstructive pulmonary disease (COPD). This study evaluated the effect of recuperating lung decoction (RLD) on inflammation and oxidative stress in rats with COPD induced by cigarette smoke and lipopolysaccharides (LPS). We used intravenous infusion of LPS combined with cigarette smoke exposure as a COPD rat model. We observed that RLD treatment increased the protein level of GSH and the ratio of GSH/GSSG but decreased 8‐OHdG and 4‐HNE in the serum. Furthermore, RLD significantly inhibited the expressions of IL‐1β, IL‐6, TNF‐α, and TGF‐β induced by cigarette smoke exposure, reduced the number of inflammatory cells in the bronchoalveolar lavage fluid, and alleviated the severity of cigarette smoke‐induced emphysema. Mechanistically, RLD treatment prevented disease through downregulation of phosphorylated‐ERK and Nrf2 expression, which regulates the production of proinflammatory cytokines. RLD treatment exerted a dramatic therapeutic effect on COPD. This study revealed a mechanism that RLD functions on the regulation of ERK signalling to inhibit inflammation.

## 1 INTRODUCTION

Chronic obstructive pulmonary disease (COPD) is a common but preventable disease characterized by persistent respiratory symptoms and airflow limitation. COPD is the fourth leading cause of death in the world and is predicted to become the third leading cause of death by 2020.^1^ More than 3 million people died of COPD in 2012, and this number accounted for 6% of all deaths globally.^2^ With the increasing prevalence of smoking in developing countries and aging populations in high‐income countries, COPD is expected to worsen over the next 30 years, and over 4.5 million annual deaths caused by COPD are predicted to occur in 2030.^3^, ^4^

Many drugs are used for COPD treatment. However, side effects are widely observed during treatment with these drugs. For instance, dexamethasone, a steroid drug, results in skin ecchymosis, high blood pressure, severe infection or peptic ulcer, and other side effects, although it is effective in treating the disease. Therefore, alternative therapeutic strategies that do not have side effects are required. In this context, herbal medicine, acupuncture, and other substitutive therapies have been examined in clinics.^5^, ^6^

Chronic inflammation and oxidant/antioxidant imbalance are important mechanisms of COPD development.^7^, ^8^ Cigarette smoking is a commonly encountered risk factor for this disease. The smoke of each cigarette contains more than 5000 chemical compounds and 10^9^ free radicals.^10^, ^11^ Numerous studies have shown that free radicals can lead to oxidative damage in the lungs. Furthermore, oxidants generate other inhalable particulates and activate inflammatory cells, including macrophages and neutrophils.

Mitogen‐activated protein kinases (MAPKs), including extracellular signal‐regulated kinases (ERKs), c‐Jun N‐terminal kinases (JNKs), and p38, exert important effects on immune responses in the lungs. MAPK signalling cascades are affected by oxidants. Among MAPKs, ERK is the most important mediator of cellular transcriptional activities, including inflammatory responses.^12^ Smoke‐derived oxidants promote the phosphorylation and activation of ERK.^13^, ^14^, ^15^ Oxidants present in cigarette smoke can also affect inflammation by regulating numerous redox‐sensitive signalling systems in the lungs. The phosphorylation and activation of MAPK (ERK1/2, p44/42MAPK) can be selectively suppressed by 2‐(2‐amino‐3‐methoxyphenyl)‐4H‐1‐benzopyran‐4‐one (PD98059), an organic compound inhibitor frequently used to block the activity of ERK1/2 protein kinase.^9^, ^16^

Nuclear factor E2‐related factor 2 (Nrf2) regulates the responses of cells to oxidative stress and lung inflammation.^17^ The level of Nrf2 is related to lung inflammatory responses. The expression of Nrf2 has been observed in COPD patients who had mild or moderate smoking histories. Mice deficient in Nrf2 show a marked increase in the number of macrophages detected in the lung lavage and tissue following cigarette smoke exposure.^18^ Meanwhile, Nrf2−/− mice show a significant attenuation in the induction of antioxidants. The reason for the decrease in antioxidants in Nrf2−/− mice is that oxidants can stimulate Nrf by activating kinases, which phosphorylate the protein and cause it to dissociate from its cytosolic inhibitor, Keap‐1. Alternatively, oxidants oxidize the cysteine residue in Keap‐1 to prevent it from binding to Nrf.^19^ Through these means, Nrf is free to translocate to the nucleus and induce the expression of antioxidant genes.^20^ Therefore, the ability of Nrf2 to regulate antioxidant and antiinflammatory response could be a mechanism for the regulation of the pathogenesis of COPD. Nrf2 is a direct substrate of ERK, which triggers Nrf2 phosphorylation and subsequent Nrf2 nuclear translocation.^21^, ^22^ ERK and Nrf2 signalling pathways are important in cigarette smoke‐induced COPD.^23^, ^24^, ^25^

In traditional Chinese medicine, the development of chronic lung disease is believed to be closely related to lung and spleen functions. Recuperating lung decoction (RLD) is a traditional Chinese medicinal compound formed under the classic theory of traditional Chinese medicine; it has demonstrated a significant prognosis effect on COPD therapy.^26^, ^27^, ^28^, ^29^ In our previous studies, we used COPD rat models^30^ to demonstrate that RLD and its derived prescriptions help scavenge oxygen‐free radicals in rat serum^31^ and improve the inflammatory state of the airways and lungs of rats with COPD.^32^ The current research aims to determine the biological function of RLD in regulating oxidant‐antioxidant imbalance and inflammation in COPD rats and reveal the possible molecular mechanism of the inhibition of oxidative stress and inflammation.

## 2 MATERIALS AND METHODS

### 2.1 Plant materials and preparation of RLD

The herbal components of RLD were purchased from Tongrentang (Tongrentang Pharmaceutical Co., Ltd., Beijing, China). All herbal components met the Chinese Pharmacopoeia Standards 2016 upon testing. The formula of RLD included 9 herbs (Table 1). which were premixed with water, decocted twice, and subjected to filtration and concentration (density is 1.25 at 60°C). Ethanol was collected from the supernatant. The extraction and preparation processes met the pharmaceutical standards proposed by Beijing University of Chinese Medicine.

**Table 1:** Composition and amount of RLD

Latin name	Ratio	Amount, g
Huang Qi (Radix Astragalus)	10	30
Fu Zi (Aconitum carmichaeli Debx)	5	15
Gui Zhi (Ramulus Cinnamomi)	3	9
Bai Zhu (Atractylodes macrocephala koidz)	2	6
Bai Shao (Radix Paeoniae Alba)	2	6
Wu Wei Zi (Fructus Schisandrae Chinensis)	2	6
Fang Feng (Saposhnikovia divaricata (Turcz.) Schischk)	2	6
Mai Dong (Ophiopogon japonicus (Linn. f.) Ker‐Gawl)	2	6
Gan Cao (Glycyrrhizae Radix)	1	3
Total		87

### 2.2 Animals

Male Sprague Dawley rats (4–6 weeks old) of SPF grade with an average weight of 160 ± 10 g (certification of rat qualification number SCXK (Beijing), 2009‐0007, Huafukang Technology Co., Ltd., Beijing, China) were used. The rats were fed in a constant 12‐hour light‐dark cycle and allowed to eat and drink freely. Before the formal experiment, adaptive feeding was implemented for a week. All feeding conditions and experimental processes performed were in accordance with the internationally accepted US Principles of Experimental Animal Use (NIH publication number 85‐23, revised in 1985) and approved by the ethics committee of the China‐Japan Friendship Hospital.

### 2.3 Modelling

The modelling method was based on our previous study with several modifications.^33^, ^34^, ^35^ To be brief, the rats were exposed to passive smoking twice daily as follows: in the morning, 5 cigarettes (Daqianmen cigarettes: tar yield 11 mg/cigarette, nicotine 0.8 mg/cigarette, CO yield 13 mg/cigarette; Shanghai Tobacco Group Co., Ltd.) were lit simultaneously for 0.5 hours for 2 times with a 10‐minute interval. The second passive smoking was carried out in the afternoon in the same manner. Continuous cigarette smoke was administered for 2 hours per day for 12 weeks. Meanwhile, the rats in the normal group were exposed to fresh air. In addition, 200 μg of lipopolysaccharides (LPS) (manufactured by US Sigma Company, article no. L2880) dissolved in PBS was administered to the airways of the rats via endotracheal intubation on days 1 and 14. Afterward, the animals were anaesthetized with 10% chloral hydrate. As a control, 0.2 mL of PBS was administered through dripping in the same manner. The rats were randomly divided into 5 groups, with 8 rats in each group. The rats in the PD98059 group were injected with 1.75 mg/kg of PD98059 (ERK1/2 inhibitor; Selleckchem Co., Ltd., Texas, USA) dissolved in 0.1% DMSO before modelling intraperitoneal injection 3 times (once a day). The model group (COPD group) was established with the description above. On the 12th week, intragastric administration of RLD (3.65 g/kg) or dexamethasone (Xianju Co., Ltd., Zhejiang, China; 0.81 mg/kg) diluted with 3 mL of PBS was conducted. An equal quantity of normal saline was administered to the normal group. This process was performed for 2 weeks. Thereafter, the rats were sacrificed, and bronchoalveolar lavage fluid (BALF), serum, and lung tissue samples were collected.

### 2.4 Enzyme‐linked immunosorbent assay (ELISA)

Tracheal intubation was performed after injecting an anaesthetic into the rats. Then, the abdomen was opened, and blood was obtained from the abdominal aorta and centrifuged at 3500r for 5 minutes to separate the serum. The concentrations of GSH, GSSG, 8‐OHdG, and 4‐HNE in the serum and the proinflammatory mediators, including tumour necrosis factor‐(TNF‐)𝛼 in the serum, TGF‐𝛽, interleukin (IL)‐6, and IL‐8 in the lung tissue, were measured with ELISA kits (R&D System, Minneapolis, MN, USA) according to the manufacturer's protocols.

### 2.5 Inflammatory cell count in BALF

BALF collected from the left lung tissues was centrifuged at 1600 × g for 15 minutes, and the supernatant was stored at −80°C. The pelleted cells were resuspended in PBS, and 1 × 10^5^ cells were subjected to cytospin centrifugation on glass slides, fixed with 100% methanol for 20 minutes, and stained with haematoxylin and eosin (H&E). A differential cell count of BALF cell pellets was performed under a light microscope at a magnification of 200× according to morphological characteristics.

### 2.6 Morphological and histopathological assessment of lung tissue

H&E staining was performed on paraffin‐embedded lung tissue sections (4 μm). The lung tissue morphology was determined through light microscopy at a magnification of 200×. The pathological score was determined according to the degree of lung tissue inflammation.^36^

### 2.7 Immunohistochemical analysis

Slices were deparaffinized with Xylol and rinsed with phosphate‐flushing fluid. Then, antigen retrieval was performed using a microwave in 10 mM of citrate buffer and pH 6. Endogenous peroxidase was blocked with 3% peroxide for 15 minutes. Primary Nrf2 (Abcam Cambridge, UK)/pERK1/2 (Cell Signalling Technology, Boston, MA) antibody was applied at 1:400 dilution overnight at 4°C. After reheating at 37°C for 1 hour, the respective secondary antibodies were added for incubation at 37°C. DAB colouring was performed, and the cell nucleus was re‐dyed with Harris haematoxylin. The samples were dehydrated until they became transparent and were sealed with neutral balsam. The slices were observed under a light microscope. Five different random views of each slice were observed. A semiquantitative analysis of integrated optical density (IOD) was conducted with HIS‐IPP (Image Pro Plus 6.0) software.

### 2.8 Detection of Nrf2 and pERK/ERK through Western blot

The protein expression in the lung tissues was evaluated through Western blot. Briefly, total protein extracts from lung tissues were separated by polyacrylamide gradient gels (12%) and transferred to polyvinylidene fluoride membranes. The membranes were incubated overnight at 4°C with primary antibodies anti‐phospho‐ERK1/2 (Cell Signalling Technology, Boston, MA), anti‐Nrf2, anti‐total ERK1/2 (Abcam Cambridge, UK), and β‐actin (1:1000 dilution). Then, the respective secondary antibodies conjugated to HRP were incubated for 1 hour, followed by 3 times of washing. The membranes were then incubated with ECL (Millipore, MA, USA) for luminescence generation. The image and grey level were analysed with Alpha Innotech and Adobe software. The protein expression level was evaluated with the β‐actin ratio.

### 2.9 Statistical analysis

Data are expressed as mean ± SD unless otherwise specified. One‐way analysis of variance (ANOVA) was performed to compare the difference using SPSS17.0 (version 17.0, SPSS, Inc., Chicago, IL, USA) software and Graphpad Prism 5.0. A statistical difference was accepted at a *P*‐value of <0.05.

## 3 RESULTS

### 3.1 RLD improves cigarette smoke‐induced lung injury

**Figure 1:**
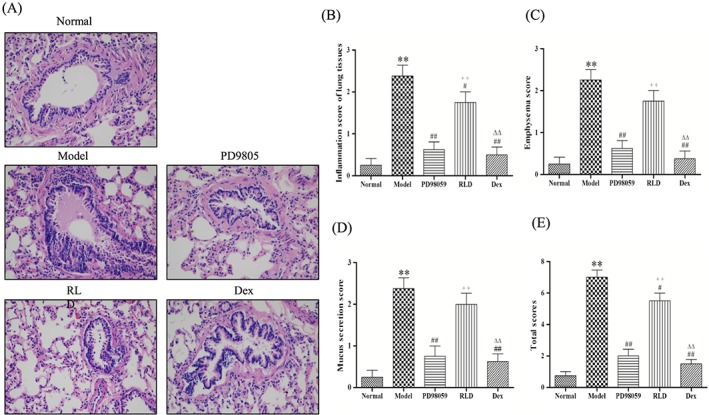
Lung injury result of lung tissues. A, H&E staining in lung tissues. B, Score for inflammation of lung tissues. C, Score for emphysema. D, Score for mucus secretion. E, Total score. Statistical analysis was performed with a non‐parametric test (Kruskal‐Wallis test), whereas group comparison was performed with the Mann‐Whitney *U* test ^**^ *P* < 0.01 compared with the normal group; ^#^ *P* < 0.05, ^##^ *P* < 0.01 compared with the model group, ++*P* < 0.01 compared with the PD98059 group; ^△△^ *P* < 0.01 compared with the dexamethasone group (*n* ≥ 6/group)

To examine the effect of RLD, a special Chinese medicinal compound preparation, we established a COPD rat model by using intravenous infusion of LPS and cigarette smoke exposure. The rats were further treated with RLD, and the disease index was assessed. Tissue morphological analysis was performed to determine the protective effect of RLD on cigarette smoke‐induced emphysema in rats. As shown in Figure 1A, exposure to cigarette smoke‐induced emphysema in rats, and bronchiole wall thickness, small pulmonary vessel wall thickness, bronchiole stenosis, and alveolar diameter increased. RLD treatment alleviated the severity of cigarette smoke‐induced emphysema, similar to the effect by dexamethasone and ERK inhibitor PD98059. Histopathological analyses on the morphological damage demonstrated that the COPD rats had a higher degree of inflammatory cell infiltration than the control group (*P* < 0.01). Inversely, the inflammatory degree in the dexamethasone group and PD98059 was alleviated (*P* < 0.01), and airway inflammation in RLD was ameliorated (*P* < 0.05), indicating that RLD functioned similarly to the inhibitors of inflammation. These results suggest that RLD significantly reduced the emphysema and inflammation induced by cigarette smoke.

### 3.2 RLD inhibits lung inflammation and ERK phosphorylation

**Figure 2:**
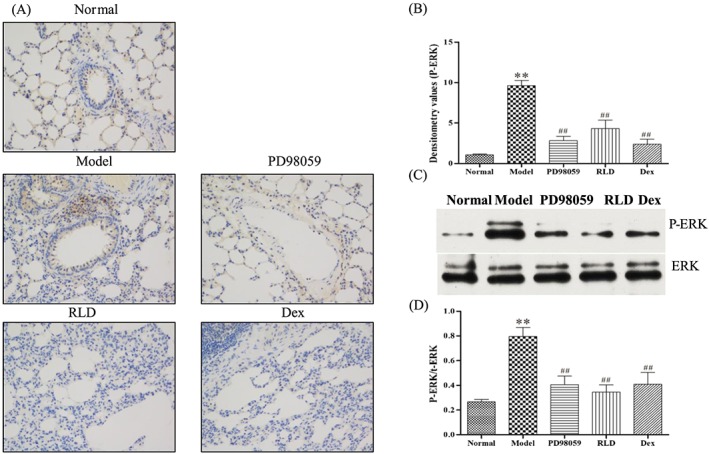
Phosphorylation levels of ERK are reduced after RLD treatment. A, Expression levels and locations of p‐ERK in lung tissues were analysed through immunohistochemistry. Brown positive expression was exhibited at the bronchial wall and alveolar septum. After RLD treatment, the positive expression of phosphorylation was reduced. B, Semiquantitative analysis of p‐ERK in lung tissues and intergroup comparison were conducted. RLD treatment effectively inhibited the phosphorylation levels of ERK. C, D, The ratios of p‐ERK/total ERK in COPD were significantly increased. The total expression levels of ERK were the same during the treatment. After RLD treatment, the phosphorylation levels of ERK were effectively inhibited. The data are expressed as mean ± SD. One‐way ANOVA was adopted for statistical analysis. The Newman‐Keuls method was used for the intergroup comparison (^**^ *P* < 0.01 compared with the normal group, ^##^ *P* < 0.01 compared with the model group; *n* ≥ 3). Abbreviation: IOD denotes integrated optical density

Cigarette smoke is a potential ERK trigger that could induce inflammation and oxidative stress through the activation of ERK signalling cascades in the lungs. To investigate whether the suppressive effect of RLD on cigarette smoke‐induced inflammation and oxidative stress is mediated through the ERK pathway, Western blot and immunohistochemical analysis were performed on the lung tissues. The results demonstrated that cigarette smoke exposure upregulated the level of phosphorylated ERK (p‐ERK). Interestingly, RLD treatment largely abolished cigarette smoke‐induced p‐ERK (Figure 2).

### 3.3 RLD inhibits the expression of Nrf2 induced by lung inflammation

**Figure 3:**
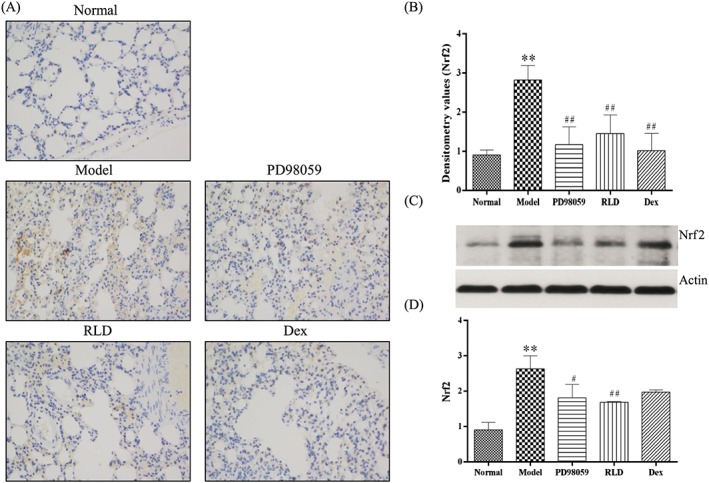
Nrf2 levels are reduced after RLD treatment. A, The expression levels and locations of Nrf2 in lung tissues were analysed through immunohistochemistry. Brown positive expression was exhibited at the alveolar septum. After RLD treatment, the positive expression of Nrf2 was decreased. B, Semiquantitative analysis of Nrf2 in lung tissues and intergroup comparison were conducted. RLD treatment effectively downregulated the levels of Nrf2. C, D, Quantitatively detected expression of Nrf2 in COPD was significantly increased. After RLD treatment, the levels of Nrf2 were effectively decreased. The data are expressed as mean ± SD. One‐way ANOVA was adopted for statistical analysis. The Newman‐Keuls method was used for the intergroup comparison (**P < 0.01 compared with the normal group, ##*P* < 0.01 compared with the model group; *n* ≥ 3). Abbreviation: IOD denotes integrated optical density

Nrf2 is an important determinant of susceptibility to cigarette smoke‐induced emphysema.^18^ To examine whether RLD plays a role in Nrf, we performed Western blot analysis and discovered that cigarette smoke exposure upregulated the protein expression of Nrf2. On the contrary, the expression of Nrf2 was downregulated after RLD treatment (Figure 3). These results suggest that RLD suppresses the expression of Nrf.

### 3.4 RLD attenuates oxidation in COPD rats induced by cigarette smoke

**Figure 4:**
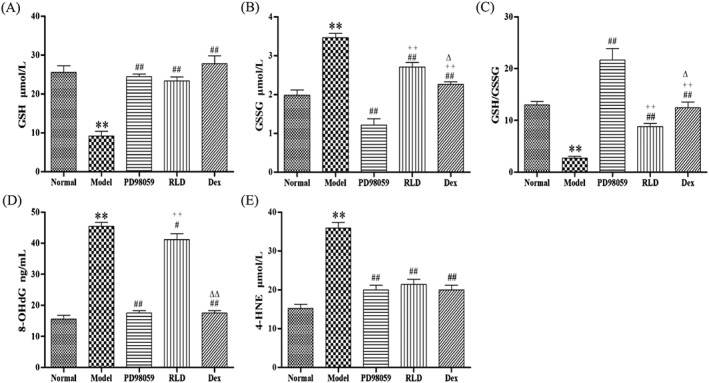
Influence of RLD on GSH, GSSG, 8‐OHdG, and 4‐HNE of COPD serum. A, The GSH concentration of the COPD group was significantly lower than that of the normal group. The GSH concentration increased after RLD treatment. B, The GSSG concentration of the COPD group was higher than that of the other groups. The GSSG level decreased after RLD treatment. C, The GSH/GSSG ratio after RLD treatment was higher than that in the COPD group. D‐E, The 8‐OHdG and 4‐HNE levels of the COPD group were higher than those of the normal group. The 8‐OHdG and 4‐HNE expression levels decreased after RLD treatment. The data are expressed as mean ± SD. One‐way ANOVA was adopted for statistical analysis. The Newman‐Keuls method was used for the intergroup comparison (**P < 0.01 compared with the normal group, ^#^ *P <* 0.05, ^##^ *P <* 0.05 compared with the model group; ++*P <* 0.01 compared with the PD98059 group, ^△^ *P <* 0.05, ^△△^ *P <* 0.01 compared with the dexamethasone group; *n* ≥ 6)

To further observe the effect of RLD on inflammation‐induced oxidant stress, we examined the level of 8‐hydroxydeoxyguanosine (8‐OHdG) and 4‐HNE, which are critical markers of DNA damage, as well as glutathione (GSH), an important antioxidant that is converted into GSSG after oxidation. The results showed that the GSH level in COPD rats was significantly lower than that in the normal group, and the GSSG level was higher (*P* < 0.01). The levels of 8‐OHdG and 4‐HNE were higher in the COPD group than in the control group. RLD treatment significantly increased the levels of GSH and the ratio of GSH/GSSG and decreased the levels of 8‐OHdG and 4‐HNE (Figure 4). As a positive control, we used PD98059 and dexamethasone to treat the rats and observed a similar effect as the RLD treatment (Figure 4). These results suggest that RLD inhibits oxidation in COPD rats.

### 3.5 RLD prevents cigarette smoke‐induced proinflammatory response in the lungs

**Figure 5:**
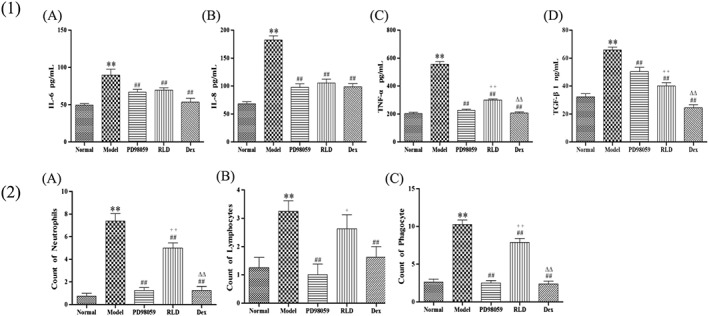
Influence of RLD on inflammation of COPD rats. (1). Effect of RLD on the level of IL‐8, IL‐6, TNF‐α, and TGF‐β. A, Level of IL‐6. B, Level of IL‐8. C, Level of TNF‐α. D, Level of TGF‐β1. (2). Inflammatory cell count in BALF. The cell numbers were counted with a hemacytometer. A, Count of neutrophils. B, Count of lymphocytes. C, Count of phagocytes. The levels were measured with a quantitative sandwich enzyme‐linked immunoassay. Data are shown as mean ± SD. Statistical analyses were conducted by 1‐way ANOVA followed by Newman‐Keuls multiple comparison test (**P < 0.01 compared with the normal group, ^##^ *P <* 0.05 compared with the model group; ++*P <* 0.01 compared with the PD98059 group, ^△△^ *P <* 0.01 compared with the dexamethasone group; *n* ≥ 6)

To observe the inflammatory response in the COPD processes by RLD, we examined proinflammatory cytokines, such as IL‐1β, IL‐6, TGF‐β, and TNF‐α, which are regulated by ERK/Nrf2. ELISA analyses indicated that IL‐6, TGF‐β, and IL‐8 in the lung tissues and TNF‐α in the serum increased in the COPD rats (Figure 5(1)). RLD significantly inhibited the expressions of the cytokines induced by cigarette smoke exposure. Simultaneously, cigarette smoke exposure increased the number of neutrophils (*P* < 0.01), lymphocytes (*P* < 0.01), and macrophages (*P* < 0.01) (Figure 5(2)), whereas treatment with RLD dramatically decreased the number of total inflammatory cells (*P* < 0.01). These results demonstrate that RLD treatment effectively inhibited the inflammatory response of the lungs.

To further investigate whether the antiinflammatory effects of RLD are mediated by ERK/Nrf2 pathways, an inhibitor for ERK (PD98059) was used before the rats were exposed to cigarette smoke. The results indicated that inhibiting ERK decreased the release of IL‐6, IL‐8, TNF‐α, and TGF‐β induced by cigarette smoke exposure. These results suggest that ERK/Nrf2 signalling regulated cigarette smoke‐induced inflammatory cytokine release, whereas the inhibitory effect of RLD on this pathway attenuated cigarette smoke‐related airway inflammation.

## 4 DISCUSSIONS

Oxidative stress and inflammation play a key pathogenetic role in COPD onset and progression.^37^ Cigarette smoking is the major risk factor for developing COPD and the major source of oxidants/reactive oxygen species (ROS) in the lungs and body.^38^, ^39^ The oxidants included in cigarette smoke can directly injure cells and tissues, inactivate defence mechanisms, and initiate inflammation. In our study, we observed that RLD effectively improved the oxidative stress response of COPD rats. As GSH, which is converted into GSSG by GSHPx, is the most abundant antioxidant, we observed its changes during the treatment of COPD rats with RLD. While the level of GSH was decreased, we also observed that the ratio of GSH/GSSG became low ratio and GSSG was increased after treatment with RLD. Correspondingly, the amounts of 8‐hydroxy‐2′‐deoxyguanosine (8‐OHdG), a marker for assessing oxidative DNA damage, and 4‐HNE, a marker of lipid peroxidation, were reduced dramatically. These results indicate that RLD improved the oxidative stress response by decreasing the oxidative DNA damage and lipid peroxidation in COPD rats.

Macrophages and neutrophils are activated during COPD for the production of proinflammatory cytokines including TNF‐α, IL‐1, IL‐6, and IL‐8. In our study, we observed that the RLD‐treated rats exhibited a reduction in inflammatory cell counts in BALF and improvement of histopathology induced by CS exposure. Correspondingly, we found that the proinflammatory cytokines were decreased by RLD treatment. At the same time, we also observed that TGF‐β was decreased in the RLD‐treated rats. As TGF‐β1 is highly expressed in the epithelium and macrophages of small airways in patients with COPD,^40^, ^41^, ^42^ we proposed that RLD might function on the macrophages and epithelium.

Endoplasmic reticulum (ER) stress is believed to contribute to the pathogenesis of COPD.^43^, ^44^ ERK, a branch in ER stress signalling pathways, activated Nrf2, which is a transcription factor and master regulator of the oxidative stress response system.^45^ Regulating the activation of the Nrf2 pathway provides cytoprotection against oxidative or electrophilic disturbances. Accumulated data indicate that Nrf2‐null cells possess lower GSH levels and higher ROS burdens than their equivalent wild‐type cells.^46^ During the development of COPD, ERK activation produces proinflammatory cytokines, such as TNF‐α, IL‐1β, and IL‐6, which aggravate airway inflammation and destroy the normal alveolar structure.^47^, ^48^Our study showed that RLD treatment can downregulate the p‐ERK protein expression. The decreased pERK may explain the role of RLD on the activation of the macrophages. Our data support a model that inhibition of ERK signalling could effectively inhibit the production of proinflammatory cytokines. On the other hand, our data showed that inhibition of ERK signalling also decreased the effect of DNA damage induced by the oxidation. Indeed, we found that RLD increased the concentration of GSH from the decreased status in the COPD rats. This increased GSH implied that RLD may relieve oxidative stress‐induced DNA damage.

Chinese herbal medicines are widely applied to treat many pulmonary diseases.^5^, ^49^, ^50^ RLD is a Chinese herbal compound produced under the basic theoretical guidance of Huangdi's Canon of Internal Medicine, a classic book on traditional Chinese medicine. This mixture of medicinal plants may have multiple targets on the therapy of COPD. Our data showed that RLD have the similar effect as Dexamethasone, a widely used antiinflammation reagent. In clinics, Dexamethasone showed the effect on the inhibition of COPD but always had certain side effects such as weight loss, diarrhoea, and peptic ulcer. From the clinical work, treatment by RLD 2‐3 Weeks, the cough and expectoration, wheezing, and other clinical symptoms have been alleviated, and spirit and physical were recovered. Some patients with lung function: FEV1/FVC% exhibited improvement. The clinical efficacy of RLD was shown. In this study and clinical work, we did not observe any side effect when we treated COPD rats and patients with RLD. Therefore, we expect that treatment with RLD in patients will show more benefits.

## 5 CONCLUSIONS

RLD relieved the oxidative stress and inflammation state of COPD. The findings indicate that RLD may have a potential role in suppressing the ERK/Nrf2 signalling pathway to prevent lung injury caused by inflammation and oxidative stress.

### Conflict of Interests

The authors declare that they have no competing interests.

### Authors' Contributions

Chunlei Li and Yue Yan acquired and analysed the data. Yanhua Kong and Longxia Gao were involved in data interpretation. Xing Zhang and Haipeng Bao performed Western blot analysis. Youlin Li and Qi Shi designed the study and drafted the manuscript. All authors read and gave their final approval for the version submitted for publication.
